# Accelerated and Synchronized Oviposition Induced by Flight of Young Females May Intensify Larval Outbreaks of the Rice Leaf Roller

**DOI:** 10.1371/journal.pone.0121821

**Published:** 2015-03-27

**Authors:** Lei Zhang, Pan Pan, Thomas W. Sappington, Weixiang Lu, Lizhi Luo, Xingfu Jiang

**Affiliations:** 1 State key Laboratory for Biology of Plant Disease and Insect Pest, Institute of Plant Protection, Chinese Academy of Agricultural Sciences, Beijing, P. R. China; 2 USDA-Agricultural Research Service, Corn Insects & Crop Genetics Research Unit, Genetics Laboratory, Iowa State University, Ames, Iowa, United States of America; CNRS, FRANCE

## Abstract

Physiological management of migration-reproduction trade-offs in energy allocation often includes a package of adaptions referred to as the oogenesis-flight syndrome. In some species, this trade-off may be overestimated, because factors like flight behavior and environmental conditions may mitigate it. In this study, we examined the reproductive consequences induced by different flight scenarios in an economically-important Asian migrant insect, *Cnaphalocrocis medinalis*. We found that the influences of flight on reproduction are not absolutely positive or negative, but instead depend on the age at which the moth begins flight, flight duration, and how many consecutive nights they are flown. Adult flight on the 1st or 2nd night after emergence, flight for 6 h or 12 h nightly, and flight on the first two consecutive nights after emergence significantly accelerated onset of oviposition or enhanced synchrony of egg-laying. The latter can contribute to subsequent larval outbreaks. However, flight after the 3rd night, flight for 18 h at any age, or flight on more than 3 consecutive nights after adult emergence did not promote reproductive development, and in some scenarios even constrained adult reproduction. These results indicate that there is a migration/reproduction trade-off in *C*.*medinalis*, but that it is mitigated or eliminated by flight under appropriate conditions. The strategy of advanced and synchronized oviposition triggered by migratory flight of young females may be common in other migratory insect pests.

## Introduction

Migration is an adaptative strategy used by many insect species to deal with spatiotemporal fluctuations in resources, both predictable and unpredictable [1]. Among migrant species, it plays an important role in modifying population dynamics, life-history strategies and host-pathogen relationships [2–5]. Energetic investments in flight organs are costly, and competition for limited internal resources may lead to physiological trade-offs with other life history components [6, 7]. Migration-mediated reproductive costs are not uncommon among insect species. For instance, in soybean aphid, *Aphis glycines*, long-distance flight decreased lifetime fecundity of parental adults and their alate offspring [8]. In several noctuid moths, including *Spodoptera exempta*, *Heliothis virescens* and *Pseudoplusia includens*, lifetime fecundity is decreased by flight [9–11]. Increased flight activity by female speckled wood butterflies, *Pararge aegeria*, can impact key life history traits such as reproductive success and longevity [12]. In the pierid butterfly, *Pieris napi*, summer generation adults had a higher dispersal capacity than those of the spring generation, but spring generation females produced more eggs than summer generation females [13]. In the firebug, *Pyrrhocoris apterus*, the preoviposition period (POP) was shorter in brachypterous than in macropterous females, although the total number of eggs did not differ [14]. In many species, flight system development and maintenance of migratory flight consume much energy, and reproductive development is suppressed during this period. This type of developmental relationship between migration and reproduction manifests as the oogenesis-flight syndrome [15–16], historically considered an obligate physiological trait of migratory insects [6, 15, 17–20].

However, not all insect species pay a net reproductive cost for migrating, and the oogenesis flight syndrome is clearly not a universal characteristic of migratory insects [2, 17, 21–23]. Indeed, some evidence suggests that migration/flight can even benefit reproduction by promoting ovarian development or shortening the POP [6, 11, 24–27]. Interestingly, the migratory strategy is more flexible than is often imagined. In some species, whether the influence of migration on reproduction is positive, negative, or neutral depends on the stage at which the individual makes the decision to migrate or remain resident. In beet armyworm, *Spodoptera exigua*, flight at different ages does not affect reproduction significantly, except for a negative effect observed after flight on day 1 of adulthood [2]. In beet webworm, *Loxostege sticticalis*, reproductive parameters for adults that experienced flight did not differ significantly from unflown moths. However, the period of first oviposition (PFO), a parameter measuring synchrony of first egg-laying in a population, for moths flown on d 3 and 5 of adulthood was shorter than that of unflown moths. The resulting increased synchrony of egg-laying can serve to increase egg and subsequent larval densities, which may contribute to development of an outbreak population [28]. Similarly, the influence of flight on reproduction in the oriental armyworm, *Mythimna separata*, differed with age at time of flight. Flight on day 1 of adulthood promoted reproduction with earlier oviposition, enhancing synchrony of first egg-laying and fecundity. However, no influence on reproduction was found after experiencing flight on day 2 to day 4, while a negative effect on reproduction appeared after flight on day 5 [26].

The rice leaf roller, *Cnaphalocrocis medinalis* (Guenée) (Lepidoptera: Pyralidae), is a major migratory insect pest of rice in Asia, East Africa and Australia. Serious outbreaks of rice leaf roller have been reported in many Asian countries in recent decades, especially in China [29–35]. It is a long-distance migratory species, with one of the greatest documented flight distances (1200 km) detected by Mark-Release-Recapture experiments [36]. In China, this species cannot overwinter north of about 30°N where the isotherm in January is about 4°C, and their reproduction suffers at temperatures > 28°C [36]. Migration by *C*. *medinalis* is an adaption to seasonal and regional variation in environmental factors, such as temperature and photoperiod [36, 37]. Reasons for outbreaks of *C*. *medinalis* not only are closely associated with appropriate environmental conditions, but also the number of immigrants in a population, which can lay eggs in very short time [36]. However, the reproductive consequences of the outbreak-promoting mechanism under different migration scenarios remains unknown in this species [38]. In this study, we designed three experiments to characterize the net effects of flight scenarios, such as the age at time of flight, flight duration, and number of nocturnal flights, on reproduction by *C*. *medinalis*. In addition, we use the period of first oviposition (PFO), a parameter we introduced previously [28], to measure synchrony of first egg-laying by a cohort of post-migratory females, to probe the mechanism behind seasonal outbreaks of immigrant populations. These results provide valuable information toward establishing effective population monitoring and forecasting capability for this species.

## Materials and Methods

### Insects

*C*. *medinalis* pupae were collected from early rice fields in Xingan County (26°18′N, 110°35′E), Guangxi Province, which had not been treated with chemicals. No specific permissions were required for taking the field sample of *C*. *medinalis*, because it is a common rice pest rather than an endangered or protected species, and the sampled field is not protected in any way. Adults emerging from these pupae were identified as emigrants based on historical observations of field populations [36]. In the laboratory, single male and female moths were paired after emergence and placed in cylindrical plastic cages (10 d × 20 h cm). They were fed with fresh 10% (v/v) honey solution (Milk vetch honey, Yishouyuan Co., China), which was changed daily throughout the experiment. All insects were reared in the insectary maintained at a constant temperature of 26±1°C, 85%-95% RH and under a photoperiod of 16L:8D [39].

### Experimental design

Three interconnected experiments were designed to characterize reproductive traits after flight. The first experiment examined the influence of flight at different days after emergence on reproduction of *C*. *medinalis*. This experiment comprised five treatments, including unflown moths (control) and moths flown at days 1, 2, 3 and 4 of adulthood. The flight test duration for all treatments was 12 h (20:00–08:00). The second experiment was designed to test the influence of different tethered flight durations of 6h, 12h, and 18h on reproduction. One-day-old adults were chosen because it has been hypothesized that *C*. *medinalis* migration begins the first night after emergence (Riley et al., 1995). The third experiment was designed to test the influence of the number of nights of flight on reproduction. There were five treatments in this experiment, including unflown moths (control) and moths flown for 1, 2, 3, or 4 nights, with duration of tethered flight opportunity of 6 h nightly (20:00–02:00). All treatments were initiated with 1-day- old adults.

### Tethered-flight technique

Flight tests were conducted on a 48-channel computer-interfaced flight mill system, which automatically records total flight distance, flight duration and average flight velocity. Each moth was tethered following the techniques used in previous work [2, 28, 40, 41]. Tested moths were anesthetized with ether, and scales at the dorsal junction of the thorax and abdomen were gently swept away. Short plastic tethers were glued to the cuticle with 502 adhesive glue (Beijing Chemical Co.). A tethered moth was attached to the arm of a flight mill. Three flight capacity parameters, flight duration, distance and speed were measured in each experiment. Ambient temperature and humidity in the flight chamber were maintained at 24±1°C and 80%-85% RH, conditions promoting maximum flight capacity of *C*. *medinalis* [41]. All flight tests began at 20:00 h and were performed under dark conditions.

### Reproductive parameters

After completion of the tethered flight, the tether was carefully removed from the moth with scissors. Each moth was paired with a male of the same age and transferred into a 1-L plastic cage, with fresh 10% honey solution provided every day until death. The number of eggs laid and mortality were recorded daily. Mating status and mating frequency were determined by the presence and number of spermatophores in the female after death. POP, PFO, lifetime fecundity, mating frequency and mating percentage were used to evaluate the effects of different flight treatments on reproduction. These parameters were determined following the methods employed in our previous studies [2, 28]. PFO describes the duration of the time window (in days) over which first oviposition occurred among individuals of a treatment group relative to the earliest case of oviposition by any moth within that group. It is a parameter for measuring synchronization of oviposition [28].

### Data analysis

All numeric values obtained from the studies are presented as means ± SE. Differences in mating percentage between treatments were compared by Chi-squared tests. Differences in all other parameters between treatments were evaluated by one-way analysis of variance. Significant differences among multiple means were determined by Tukey’s HSD test. All statistical analyses were performed using SAS version 9.1 software.

## Results

### Influence of flight on POP

**Fig 1:**
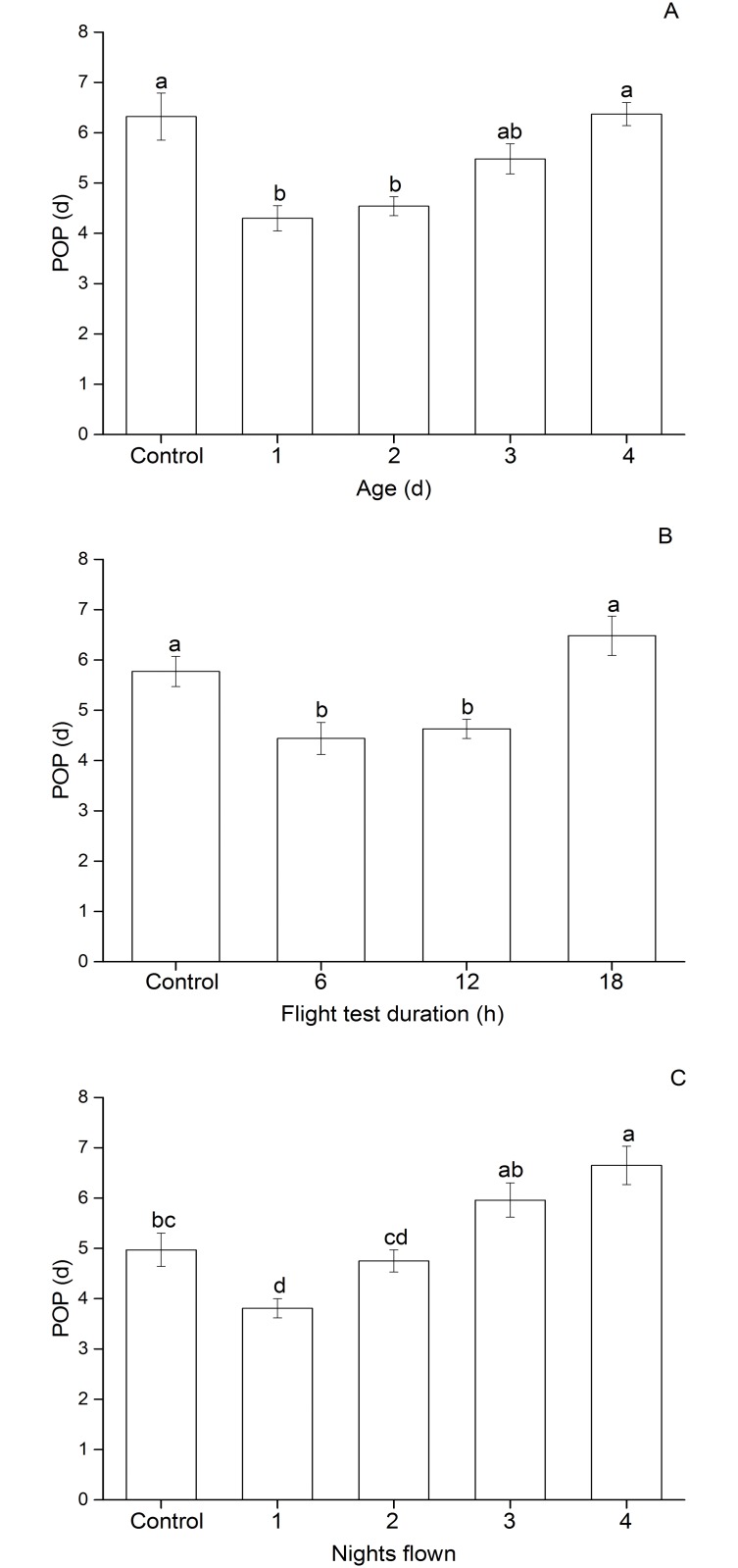
Preoviposition period (POP) of adult *C. medinalis* that experienced (A) a 12-h tethered-flight test at different ages, (B) different flight test durations at 1 day of age, and (C) flight tests of 6-h duration on successive nights beginning at 1 day of age. Data are presented as mean ± SEM. Bars sharing the same letter are not significantly different at 5% level by Tukey’s HSD test. Sample sizes for each treatment in panel A are 25, 23, 26, 23 and 19; in panel B are 31, 27, 27 and 29; and in panel C are 31, 33, 31, 27 and 30, from left to right, respectively.

POP of *C*. *medinalis* was significantly affected by the age at which the female was flown (*F*_4, 111_ = 9.35, *P* < 0.0001), ranging from 4.30 to 6.32 days (Fig. 1A). The POPs of females flown at d 1 and d 2 were significantly shorter than those of the control and moths flown at d 4. However, females flown at d 3 and d 4 did not oviposit significantly earlier than control moths (Fig. 1A). Different durations of flight tests also significantly affected POP of 1-day-old females (*F*_3, 110_ = 9.59, *P* < 0.0001), which ranged from 4.44 to 6.48 days (Fig. 1B). POPs of females following 6 h and 12 h of tethered flight were significantly shortened more than 1 d compared to those of moths tested for 18 h or the control group. The mean POP of females in the 18 h flight treatment did not differ significantly from that of the control (Fig. 1B). POP was also significantly influenced by the number of nights of 6-h flight tests (*F*_4, 143_ = 14.31, *P* < 0.0001, Fig. 1C). One night of tethered flight significantly accelerated female reproductive development by more than 1 day compared to the unflown control moths. No significant difference was observed in POP between the control group and females flown for 2 and 3 nights. However, females flown for 4 nights showed a significant delay in reproductive development (Fig. 1C).

### Influence of flight on general reproductive traits

Flight ages significantly influenced mean lifetime fecundity among all treatments, which ranged from 34.53 to 118.60 eggs (*F*_4, 111_ = 3.66, *P* = 0.0077) (Table 1). Fecundity was significantly less for moths in the d-3 and d-4 treatments compared to the controls. Mean oviposition period (*F*_4, 111_ = 19.83, *P* < 0.0001) of females flown at any age except 1 day were significantly less than the unflown controls (Table 1). Female longevity did not differ with age, but it significantly declined compared to unflown controls (Table 1). There were no significant differences among any treatments for mating percentage (*χ* ^2^ = 2.37, *df* = 4, *P* = 0.67) or frequency (*F*_4, 111_ = 0.58, *P* = 0.68) (Table 1). Lifetime fecundity, oviposition period, mating frequency and mating percentage were not negatively affected by flight tests of 12 h or less (Table 2). However, a flight test of 18 h did negatively affect all of these reproductive traits compared to the control group. In contrast, female longevity was not significantly affected by any of the flight treatments except for a significant decrease in the 12-h flight group (Table 2). The number of nights flown also significantly influenced lifetime fecundity, mating frequency, and mating percentage, but this was due mainly to a significant decrease in the 4-nights group (Table 3). Female longevity was not significantly affected by the number of nights flown.

**Table 1:** Life-time fecundity, ovipositon period (number of days from first oviposition through the last day), mating percentage, and longevity of adult *C*. *medinalis* after experiencing a 12-h flight test at different ages of adult life.

Age (d) at flight	Lifetime fecundity	Oviposition period (d)	Mating frequency	Mating percentage (%)	Female Longevity (d)
Control	118.60±32.01 a (25)	5.52±0.40 a (25)	0.36±0.10a (25)	36.00	13.76±0.53 a (25)
1	89.57±11.35 ab (23)	4.52±0.24 ab (23)	0.30±0.10a (23)	30.43	9.57±0.37 b (23)
2	92.54±9.76 ab (26)	4.42±0.19 b (26)	0.31±0.09a (26)	30.77	9.81±0.36 b (26)
3	52.13±5.13 b (23)	4.00±0.29 b (23)	0.26±0.09a (23)	26.09	10.22±0.38 b (23)
4	34.53±4.49 b (19)	2.58±0.26 c (19)	0.16±0.09a (19)	15.79	9.26±0.46 b (19)

*Data are presented as mean ± SE. Number in parentheses is the corresponding sample size. In each column, data sharing the same letter are not significantly different at the 5% level by Tukey’s HSD test. The mating percentages between each flown group and the unflown control group are not significantly different, as determined by a Chi-square test (*χ* ^2^ = 2.37, *df* = 4, *P* = 0.67).

**Table 2:** Lifetime fecundity, ovipositon period, mating percentage, and longevity of adult *C*. *medinalis* after experiencing different flight test durations as 1-d-old adults.

Flight test duration (h)	Lifetime fecundity	Oviposition period (d)	Mating frequency	Mating percentage (%)	Female longevity (d)
Control	124.42±19.23 a (31)	5.75±0.45a (31)	0.42±0.09 a (31)	41.94 a	12.39±0.61a (31)
6	113.89±16.57 a (27)	5.59±0.42a (27)	0.44±0.10 a (27)	44.44 a	11.11±0.48ab (27)
12	82.30±12.44 ab(27)	5.63±0.35a (27)	0.22±0.08 ab (27)	22.22 ab	10.48±0.36 b (27)
18	36.00±7.23 b (29)	3.83±0.39b (29)	0.07±0.05 b (29)	6.90 b	11.14±0.52ab (29)

*Data are presented as mean ± SE. Number in the parentheses is the corresponding sample size. Data in a column sharing the same letter are not significantly different at 5% level, as determined by Tukey’s HSD test. The treatments significantly affected mating percentage, as determined by Chi-square test (*χ* ^2^ = 13.39, *df* = 3, *P* = 0.0039).

**Table 3:** Fecundity, ovipositon period, mating frequency, mating percentage, and longevity of female *C*. *medinalis* after experiencing flight tests of 6-h duration on successive nights beginning at 1 day of age.

Nights flown	Lifetime fecundity	Oviposition period (d)	Mating frequency	Mating percentage (%)	Female longevity (d)
Control	155.90±24.56 a (29)	5.89±0.45a (29)	0.52±0.12a (29)	44.83	11.52±0.52 a (29)
1	185.90±30.29 a (31)	6.51±0.57a (31)	0.42±0.09ab (31)	41.93	11.13±0.65 a (31)
2	132.06±16.86 ab (32)	6.78±0.49a (32)	0.31±0.08ab (32)	31.25	12.09±0.55 a (32)
3	131.37±25.19 ab (27)	6.59±0.67a (27)	0.33±0.09ab (27)	33.33	13.26±0.68 a (27)
4	66.34±10.99 b (29)	6.31±0.59a (29)	0.14±0.06b (29)	13.79	13.52±0.67 a (29)

*Data are presented as mean ± SE. Number in parentheses is the corresponding sample size. Data in a column sharing the same letter are not significantly different at 5% level, as determined by Tukey’s HSD test. The mating percentage between the treatments was tested by the Chi-square test showing no signicantly different from each other (*χ* ^2^ = 7.03, *df* = 4, *P* = 0.13).

### Influence of flight on PFO

**Fig 2:**
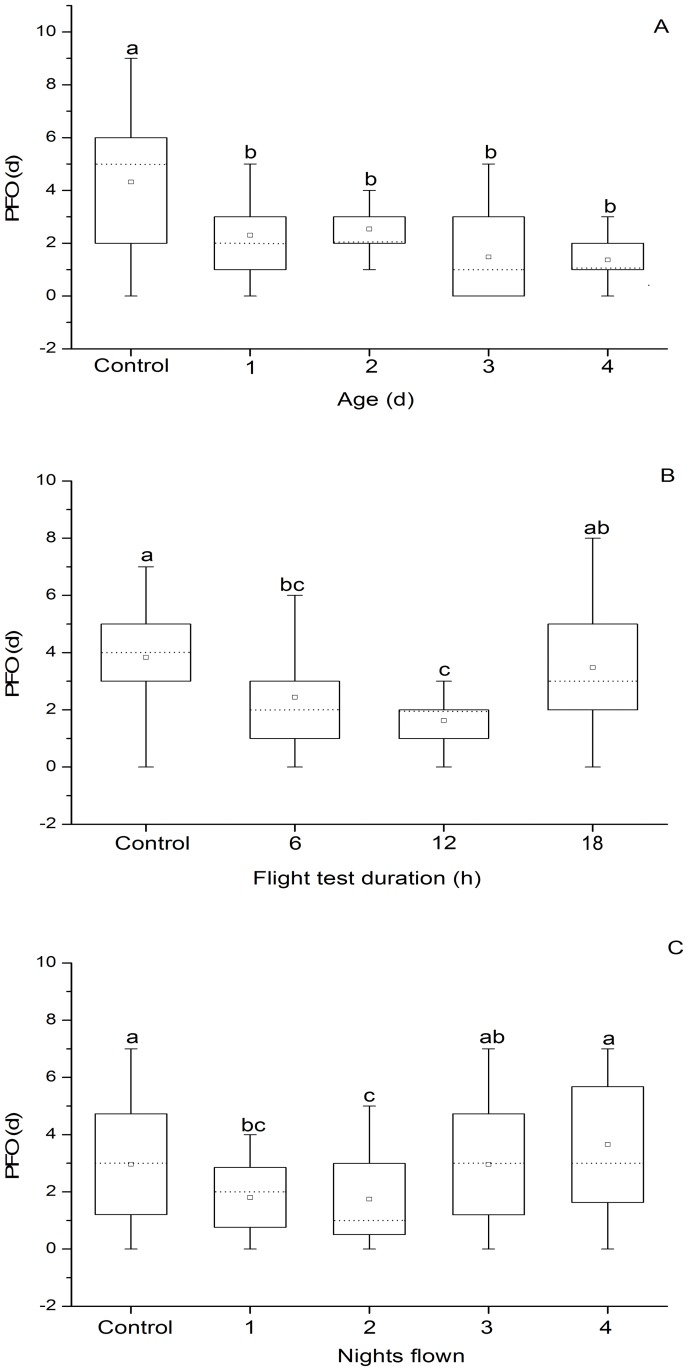
Period of first oviposition (PFO) of adult *C. medinalis* that experienced (A) a 12-h tethered-flight test at different ages, (B) different flight test durations at 1 day of age, and (C) flight tests of 6-h duration on successive nights beginning at 1 day of age. Data are presented (from top to bottom in each of the box-and-whiskers plots) as the maximum (-), upper quartile (—), mean (□), median (---), lower quartile (—), and the minimum (-). Means with the same letters in each panel are not significantly different at 5% level by Tukey’s HSD test. Sample sizes for each treatment in panel A are 25, 23, 26, 23 and 19; in panel B are 31, 27, 27 and 29; and in panel C are 31, 33, 31, 27 and 30, from left to right, respectively.

PFO of female *C*. *medinalis* was significantly affected by flight age (*F*_4, 111_ = 13.47, *P* < 0.0001) (Fig. 2A). All treatment groups experiencing tethered flight showed significantly shorter PFO than the unflown control group, but did not differ significantly from each other, suggesting the timing of initial oviposition was more synchronous among moths flown at any age tested than among those not flown. Among 1-d-old moths, mean PFO was significantly affected by flight test duration. (*F*_3, 110_ = 10.75, *P* < 0.0001) (Fig. 2B). Mean PFO of females from the 6-h and 12-h flight test periods was significantly less than that of the unflown control moths, but were not significantly different from each other. Thus, the 18-h flight test did not shorten the PFO of the female, but tended to prolong the window of oviposition initiation, and was significantly longer than 12-h test moths. In other words, the longest flight duration tested did not intensify synchrony of oviposition, in contrast to the case of females flown for ≤ 12 h. The number of nights females were flown also significantly influenced the PFO (*F*_4, 143_ = 8.10, *P* < 0.0001) (Fig. 2C). Females flown for 1 or 2 nights oviposited significantly earlier, with mean PFO 1.16 d and 1.22 d less than the controls, respectively. However, no significant differences were observed among the unflown controls and those flown for 3 or 4 nights. Therefore, initiation of oviposition was more synchronous among moths flown on one of the first two nights after emergence than among unflown controls or moths flown on nights 3 or 4.

## Discussion

### Possible migratory flight mode in *C*. *medinalis*

Migration, as a component of an insect's life history, plays a significant role in population maintenance and evolution, and by implication must provide reproductive benefits over remaining sedentary, at least on average [2, 3]. The reproductive consequences of *C*. *medinalis* moths flown at different ages suggest that migratory flight may be most advantageous on the first or second night after adult emergence. A significant decrease of POP was observed in adults flown on d-1 or d-2 after emergence compared to the control and adults flown on d-4, and their lifetime fecundity was not significantly different from that of the control. In addition, their PFOs were significantly shorter than that of the control. Although POP for the adults flown on d-3 or d-4 did not differ from that of the control group, their lifetime fecundity was significantly less. Thus, a *C*. *medinalis* female pays a reproductive cost if it begins its first flight after d-2. This deduction is consistent with our previous results from the active flight monitoring system that showed greatest flight propensity on the first day after emergence [42], and also is consistent with field observations [34, 43, 44]. Together, our results suggest that migratory flight of *C*. *medinalis* is initiated on the night of adult emergence or at dusk the following two nights.

This is similar to the case of the gregarious form of African armyworm, *Spodoptera exempta*, which initiates migratory flight on the first night after eclosion or at dusk of the following night [45]. However, it differs from the beet webworm, *Loxostege sticticalis*, which may spend one or two days after emergence in their natal habitat garnering supplemental nutrition to allow full development of the flight system before taking off [28].

The reproductive consequences of 1-d-old adults flown for different times suggest that it may be detrimental for *C*. *medinalis* to engage in migratory flight for more than 12 h within one day. Lifetime fecundity of moths tested for 18 h significantly decreased, and POP and PFO were significantly longer than 12-h test moths. This deduction is supported by field radar observations, which demonstrated *C*. *medinalis* moths ascend at dusk and terminate flight before the following dawn [44, 46]. However, it is presumed that flight duration may extend beyond 12 h when migration occurs over water, given that migrating *C*. *medinalis* moths have been caught on ships in the East China sea during the daytime [47]. We postulate that moths flying under these conditions contribute less to larval outbreaks in the receiving areas because of the reproductive cost they suffer.

Finally, it is also detrimental to *C*. *medinalis* to engage in migratory flight for more than 3 nights. Negative reproductive consequences gradually accumulated if adults were flown for more than 3 nights. In contrast, adults flown for 1 or 2 nights exhibited a significantly shortened PFO and POP, and their lifetime fecundity was not significantly decreased compared to unflown controls.

### *C*. *medinalis* migratory flight pattern minimizes reproductive trade-offs

A trade-off between flight and reproduction in migratory insects when both traits are energetically costly constitutes typical evidence supporting evolution of migration as a life history trait [6]. Such a trade-off is usually manifested as the “oogenesis-flight syndrome”. However, the oogenesis-flight syndrome is not a universal characteristic of migratory insects [2, 17, 21–23]. In female large aspen tortrix, *Choristoneura conflictana*, energy expended during tethered flight was negatively correlated to fecundity, but there was no direct effect of flight on commencement or duration of mating and egg production [48]. Indeed, in some migratory insect species, flight promotes adult reproduction [26]. In the case of the migratory grasshopper, *Melanoplus sanguinipes*, individuals that engaged in flight oviposited earlier and with greater output than did residents or unflown controls [6, 27, 49]. Lifetime fecundity of the Glanville fritillary butterfly, *Melitaea cinxia*, was higher in more dispersive females than in less dispersive individuals [50]. In the wing polymorphic cricket, *Gryllus texensis*, mating ability and ovary weight of the flight-capable morph is generally poorer than that of the short-winged morph, but this disparity is removed by a short period of flight [51, 52]. It is possible that the trade-off between flight and reproduction in most migrant insect species could be mitigated by an appropriate developmental and behavioral pattern of flight, which differs depending on the species.

In this study, POPs of moths flown for 12 h on d-1 or d-2 were significantly shortened, while those of moths flown on subsequent days were not, suggesting that early flight of adults promotes ovarian development. Similar results were observed in *Mythimna separata* and *M*. *sanguinipes* [26, 49]. However, different results were observed in *L*. *sticticalis* and *S*. *exigua*, where flight on the first day after eclosion significantly restrained ovarian development [2, 28]. One-day-old *C*. *medinalis* flight-tested for 18 h exhibited a prolonged POP and a significant decrease in lifetime fecundity, demonstrating a notable reproductive cost. Interestingly, moths showed a significant delay in oviposition initiation (i.e., increased POP) compared to unflown controls if they were flown more than three nights, but a shortened POP or no difference was observed if flown within 3 nights of emergence.

Increased POP, and decreased lifetime fecundity and mating status or frequency, are often considered major reproductive costs in other insects engaging in migratory flight. Such costs were observed in our study when *C*. *medinalis* were flown at d-3 or d-4, or flown more than 12 h. Females flown more than 3 nights experienced both increased POP and decreased lifetime fecundity. Furthermore, decreased mating success was observed for adults flown more than 12 h or 3 nights. Together, our results suggest that migratory *C*. *medinalis* moths are most likely to take off on the first night after eclosion, and fly for 12 h or less each night over the first two nights. Such a pattern will result in little or no reproductive cost for the migrating moth.

All experimental *C*. *medinalis* females were provided with 10% fresh honey solution ad lib, reflecting observations of females in the field. Newly emerged adults feed on plant nectar or honeydew, and the supplementary nutrition is necessary for reproduction and longevity [53, 54]. Supplementary nutrition can mitigate the trade-off between flight and egg reproduction in some migratory insects. Gunn et al. (1989) showed that the effect of flight on fecundity of *S*. *exempta* depends on whether the insects were provided with nutrients after flight [10]. In milkweed bugs, *Oncopeltus fasciatus*, adult food consumption is also important in minimizing the negative effects of flight on egg production [55]. Therefore, the effect of food imbibed after flight on reproductive characteristics in *C*. *medinalis* should be investigated further.

### PFO and its role in outbreaks in *C*. *medinalis* after migration

The novel parameter PFO can be examined for its possible role in larval outbreaks of *C*. *medinalis* after migration. Previously, we documented that migratory flight in *L*. *sticticalis* induced a tightened time window of onset of oviposition after flight (i.e., decreased PFO), and that the subsequent increase in larval density can directly result in a population outbreak [28]. We also speculated that the strategy of synchronized oviposition may be common in other migratory insect pests, such as locust [56] and armyworm [26]. This hypothesis is supported by our laboratory study of *C*. *medinalis* (Fig. 2). The PFO of *C*. *medinalis* adults varied with flight age and flight duration. In general, PFOs decreased after flight in all flight-age treatments. PFOs were also significantly shortened when 1-d old moths were flown for 6 or 12 h, but not for18 h. Therefore, we conclude that the PFO of adult *C*. *medinalis* decreases if they engage in a flight of appropriate duration (< 12 h) regardless of age of first flight. Furthermore, PFOs were also significantly shortened when moths flew on 2 nights. Decreased PFO triggered by migratory flight will result in more-synchronized oviposition and intensified larval density in an area receiving a pulse of migrants. Enhanced larval density can lead to outbreak populations of *C*. *medinalis*. This mechanism could potentially play a significant role in causing or enhancing outbreaks in this species.
